# The genera *Erhaia* and *Tricula* (Gastropoda, Rissooidea, Amnicolidae and Pomatiopsidae) in Bhutan and elsewhere in the eastern Himalaya

**DOI:** 10.3897/zookeys.929.49987

**Published:** 2020-04-22

**Authors:** Edmund Gittenberger, Pema Leda, Jigme Wangchuk, Choki Gyeltshen, Björn Stelbrink

**Affiliations:** 1 Naturalis Biodiversity Center, P.O. Box 9517, NL-2300 RA Leiden, The Netherlands Naturalis Biodiversity Center Leiden Netherlands; 2 National Biodiversity Centre, Serbithang, Thimphu, Bhutan National Biodiversity Centre Thimphu Bhutan; 3 Ugyen Wangchuck Institute for Conservation and Environmental Research, Bumthang, Bhutan Ugyen Wangchuk Institute for Conservation and Environmental Research Bumthang Bhutan; 4 Department of Animal Ecology & Systematics, Justus Liebig University, Heinrich-Buff-Ring 26-32 IFZ, D-35392 Giessen, Germany Zoological Institute, Department of Environmental Sciences, University of Basel Basel Switzerland; 5 Zoological Institute, Department of Environmental Sciences, University of Basel, Vesalgasse 1, 4051 Basel, Switzerland Department of Animal Ecology & Systematics, Justus Liebig University Giessen Germany

**Keywords:** *
Erhaia
*, *
Tricula
*, 16S rRNA, taxonomy, distribution, Nepal, India, Bhutan

## Abstract

Shells of the Rissooidea species that are known from Bhutan are characterized. *Tricula montana* is reported from that country for the first time. Two *Erhaia* species from Bhutan are described as new to science, viz. *E. jannei***sp. nov.**, and *E. pelkiae***sp. nov.**, The holotypes of the *Erhaia* species that were described from Nepal are figured with photographs for the first time and compared with the congeneric taxa from Bhutan and India. *Erhaia nainitalensis* is considered a senior synonym of *E. chandeshwariensis*. An identification key is presented for the *Erhaia* species of the Himalayan foothills.

## Introduction

The rissooidean gastropods that are widespread over the globe have a confusing history of taxonomic rearrangements that follow the increasing amount of morphological and molecular data, the ongoing methodological refinements in cladistics and the wealth of more or less conflicting speculations in the phylogeography of the taxa. The species of the Rissooidea Gray, 1847 from Bhutan, Nepal and northern India that are dealt with here, are classified in two genera that belong to different families, viz. the family Amnicolidae Tryon, 1863, with the genus *Erhaia* Davis & Kuo, 1985 and the family Pomatiopsidae Stimpson, 1865, with the genus *Tricula* Benson, 1843. Some species of these two genera are intermediate hosts for Platyhelminthes that are medically significant since they may transmit the human lung fluke *Paragonimus* Braun, 1899 or human *Schistosoma* Weinland, 1858 (Liu et al. 2014).

These species are characterized by minute shells that cannot always be recognized easily from descriptions and identified because of the limited number of diagnostic characters and the fact that many conchological character states that are used in the literature cannot be strictly quantified. The general shape of the shell and the form of the aperture may be described as ovoid, conical, subcylindrical, squat, or with another term of that kind. The convexity of the whorls and the depth of the suture are equally difficult to describe unequivocally. The surface of the shells is often heavily encrusted, so that the microsculpture of the proto- and teleoconch cannot always be recognized. Despite all this, an attempt is made here to characterize the genus conchologically.

The anatomy of these micro-snails cannot easily be investigated, so that DNA sequencing has become a promising tool to investigate the systematics of the Rissooidea. The classification of two *Erhaia* species from Bhutan is based now on DNA data, whereas a third species from that country is considered congeneric by reason of conchological and ecological similarity. Three nominal species of *Erhaia* from Nepal and one species from nearby northern India are compared with the Bhutanese taxa in more detail because of their joint occurrence in springs and brooklets of the southern foothills of the Himalaya. Photographs of *Tricula montana* from Bhutan, of the holotypes of the two new Bhutanese *Erhaia* species, and of a specimen of the third Bhutanese *Erhaia* species from its type locality, are provided together with photographs of the three Nepalese nominal species of *Erhaia*, that are published here for the first time.

## Material and methods

**Figure 1.**
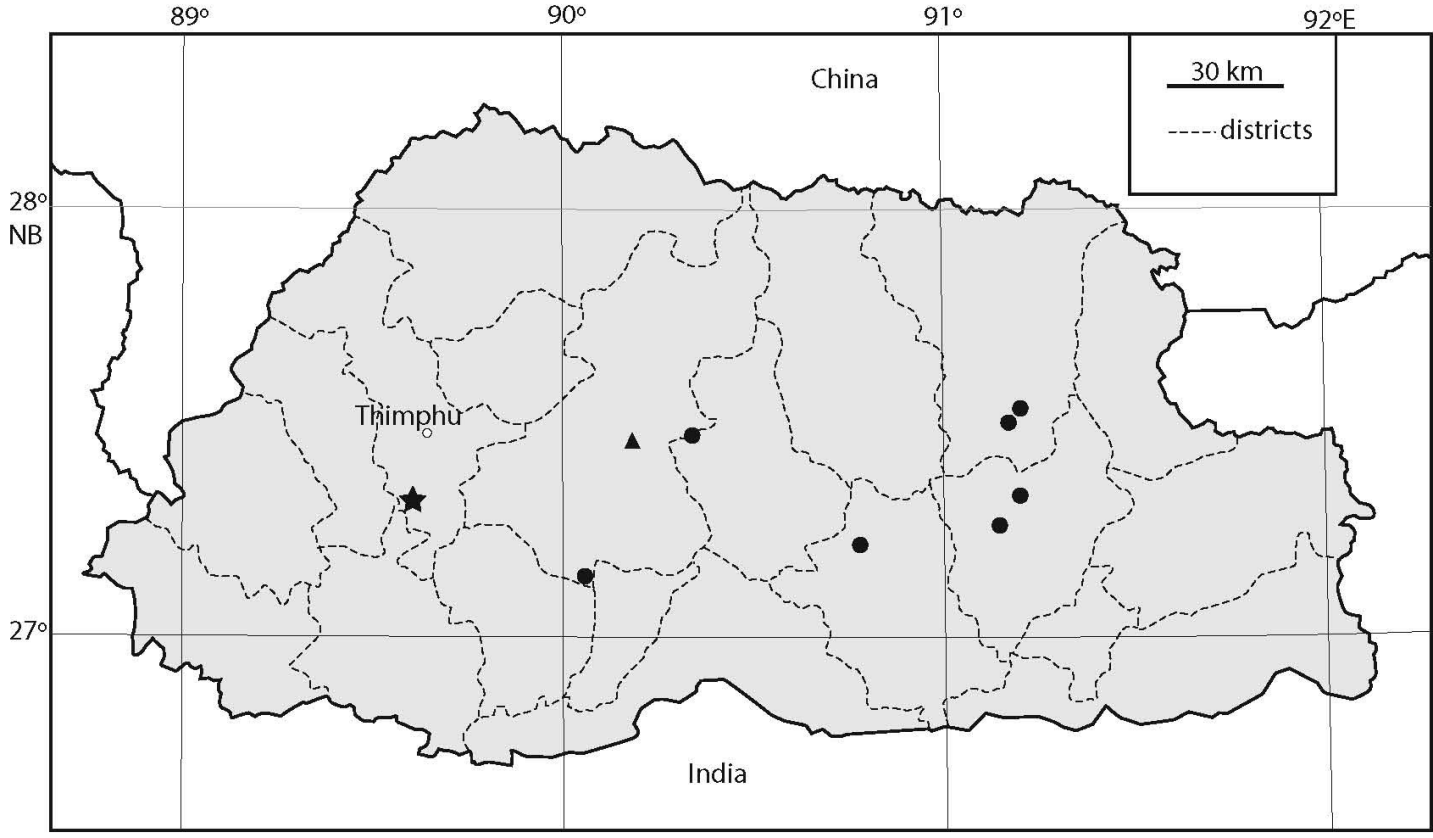
Records of the sympatric *Erhaia jannei* and *E. pelkiae* (star), *E. wangchuki* (triangle), and *Tricula montana* (dots) in Bhutan.

**Figures 2–10.**
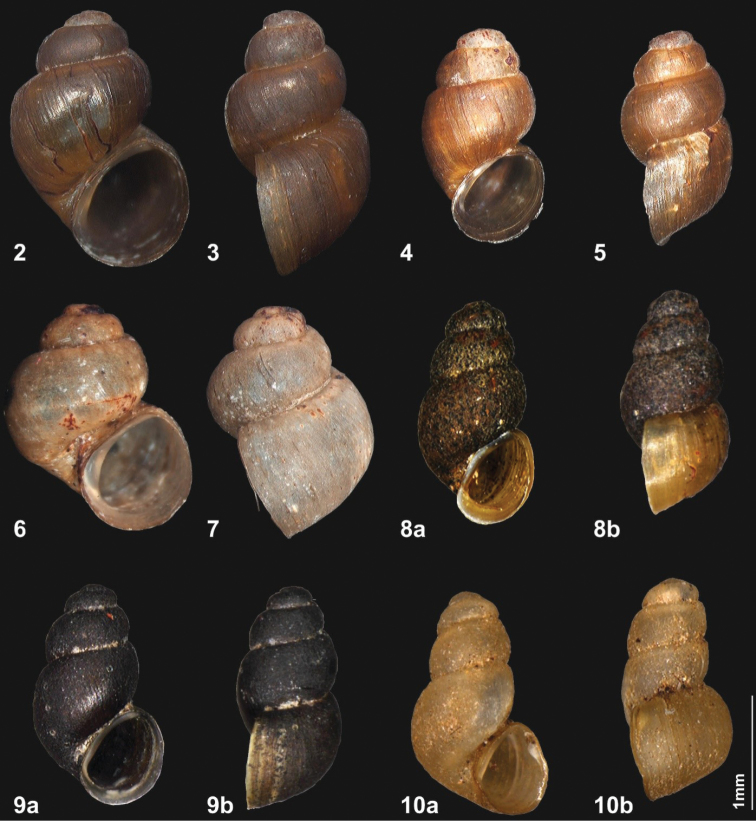
*Erhaia jannei* sp. nov., holotype (**2** H 2.25 mm) and paratype (**3** H 2.02 mm). Bhutan, district Thimphu, W of Geneykha, brooklet with water powered prayer wheel, 2750 m a.s.l. Photos by J. Goud *Erhaia pelkiae* sp. nov., holotype (**4** H 1.86 mm) and paratype (**5** H 1.86 mm). Bhutan, district Thimphu, W of Geneykha, brooklet with water powered prayer wheel, 2750 m a.s.l. Photos by J. Goud *Erhaia wangchuki* Gittenberger, Sherub & Stelbrink, 2017. Shells from the type locality (**6** H 1.98 mm) (**7** H 2.03 mm); NBCB 1072. Bhutan, district Wangdue Phodrang, Gangchhu, Gangzetem brooklet, 2883 m a.s.l. Photos by J. Goud **8***Erhaia banepaensis* Nesemann & S. Sharma, 2007, holotype (H 1.95 mm); NHMW 103319. Nepal, Central Zone, Kavre district, small forest stream, left tributary of the Chandeswari Khola upstream from Chandeshwari at Banepa. NHMW **9***Erhaia nainitalensis* Davis & Rao, 1997, holotype of *E. chandeshwariensis* Nesemann & S. Sharma, 2007 (H 1.94 mm); NHMW 103315. Nepal, Central Zone, Kavre district, small forest stream, left tributary of the Chandeswari Khola upstream from Chandeshwari at Banepa. NHMW **10***Erhaia sugurensis* Nesemann, Shah & Tachamo, 2007, holotype (H 1.95 mm); NHMW 104172. Nepal, Central Zone, Lalitpur district, Godawari, upper reaches of Sugure Khola forest stream, 1700 m a.s.l. NHMW.

Four species of minute snails were collected in spring areas and in a brooklet in Bhutan (Fig. 1). *Tricula montana*, *Erhaia wangchuki* and two undescribed *Erhaia* species could be recognized conchologically. Representative shells of these four species were photographed by Björn Stelbrink (Figs 16, 17) with a digital microscope system (KEYENCE VHX-2000; KEYENCE Corp., Itasca, IL, USA) and Mr Jeroen Goud (Figs 2–7) with a ZEISS SteREO Discovery.V20. Specimens of *T. montana* and of one of the two new *Erhaia* species were used for DNA analysis. Only two specimens of the third Bhutanese *Erhaia* species were available and these were kept as dry shells.

Photographs of the holotypes of the three *Erhaia* species that were described from Nepal and illustrated with drawings only were made with a Nikon SMZ25 stereomicroscope by Ms Sara Schnedl and provided for study by Ms Anita Eschner (both Museum of Natural History, Vienna, Austria). The only *Erhaia* species that is known from the Himalayan foothills in India is compared with the species from Bhutan and Nepal on the basis of its detailed description and photographs that are available in the literature. An identification key for the *Erhaia* species in the study area, using shell characters, is provided.

The standard CTAB protocol for molluscs was used for the DNA lab isolation (Winnepenninckx et al. 1993). In addition to the 16S rRNA fragment (c. 535 bp; using standard primers by Palumbi et al. 1991), the mitochondrial COI gene was also amplified (658 bp; standard primers by Folmer et al. 1994). The final genetic dataset mainly comprised sequences from the study by Liu et al. (2014) and additional sequences for *Tricula* that were obtained from Guan et al. (2008). Uncorrected genetic p-distances for 16S rRNA and COI were calculated using MEGA 7.0.20 (Kumar et al. 2016).

The following abbreviations are used: B = shell breadth; H = shell height; NBCB = National Biodiversity Centre, Serbithang, Thimphu, Bhutan; NHMW = Naturhistorisches Museum, Wien, Austria.

## Systematics

### Superfamily Rissooidea Gray, 1847

#### Key to the *Erhaia* and *Tricula* species from Bhutan

**Table:** 

1	Aperture ovoid; palatal side curved and gradually passing into the basal side	*** Erhaia ***
–	Aperture triangular with broadly rounded edges; palatal side straight	*** Tricula ***

#### Family Amnicolidae Tryon, 1863

##### Genus Erhaia

Taxon classificationAnimaliaLittorinimorphaAmnicolidae

Davis & Kuo, 1985

7606A5B8-245F-52F7-ADF3-9BA5978B68C1

###### Type species by original designation.

*Erhaia daliensis* Davis & Kuo, in Davis et al. 1985.

###### Shells.

The shells vary from conical to more or less ovoid, rarely with a flaring final part of the body whorl. The apex is flattened as in the European amnicolid genus Bythinella Moquin-Tandon, 1856, because of the very low spiral of the protoconch. The peristome is continuous and may be more or less protruding. The parietal and the columellar side of the aperture are about equally long and the regularly curved palatal side of the aperture gradually passes into the basal side, forming a single, regularly curved border. Bythinella cannot be distinguished from Erhaia conchologically, but in some Erhaia species from China the columella has one or two spiral lamellae, that are not known from Bythinella. In Erhaia the protoconch may have spiral striae, which have not been described for any of the Bythinella species.

###### Distribution.

The genus Erhaia is mainly known from China, where it has been recorded with various species from the province of Yunnan in the west to the provinces of Hunan, Hubei and Fujian in the east (Davis et al. 1985; Davis and Kang 1995; Davis and Rao 1997; Wilke et al. 2000, 2001; Liu et al. 2014). One species was described from northern India (Davis and Rao 1997), three from Nepal (Nesemann et al. 2007) and one from Bhutan (Gittenberger et al. 2017a). Here we deal with the systematics of only the species occurring in the southern Himalayan foothills in Bhutan, India, and Nepal.

#### Key to the *Erhaia* species from Bhutan, Nepal and northern India

**Table:** 

1	Final half of the body whorl conspicuously flaring; whorls of the spire flattened	***E. sugurensis*** (Fig. 10)
–	Final half of the body whorl not flaring; whorls convex	**2**
2	Aperture measuring half the total shell height or more	**3**
–	Aperture measuring less than half the total shell height	**4**
3	Shell conical, umbilical chink wide	***E. wangchuki***
–	Shell ovoid, umbilical chink narrow	***E. jannei* sp. nov.**
4	Parietal border of the aperture attached to the body whorl	**5**
–	Parietal border of the aperture touching the body whorl or free	***E. nainitalensis***
5	Spire turreted; shell base straight in side view	***E. banepaensis***
–	Spire ovoid; peristome widened basally and shell base concave in side view	***E. pelkiae* sp. nov.**

### *Erhaia* in Bhutan

#### Erhaia jannei

Taxon classificationAnimaliaLittorinimorphaAmnicolidae

Gittenberger & Stelbrink sp. nov.

C16E201E-F7AA-5B7B-B2E1-D9D2C4050BD6

http://zoobank.org/3CEAF442-E7C8-4699-B291-882EE902D7D7

Figs 2 , 3 , 11

Erhaia sp. Gittenberger et al. 2017a: 25, fig. 3; 2017c: 900, 903, fig. 8.

##### Material examined.

***Holotype*.** (Fig. 2) Bhutan • District Thimphu, c. 5 km E of Chhuzom, W of Geneykha; in brooklet with a prayer wheel along the road; 2750 m a.s.l.; 27°18'43"N, 89°36'10"E; E. Gittenberger, Choki Gyeltshen & Pema Leda leg. 25.X.2018; NBCB 1057. ***Paratypes*.** (Fig. 3) 6 shells and 2 animals in ethanol 70%; same data as for holotype; NBCB 1058.

##### Diagnosis.

Shell large for the genus (H > 2mm), ovoid, with a relatively large aperture.

##### Shell.

Shell obliquely ovoid, with 3½-4 convex, shouldered whorls that are separated by a deep suture; clearly higher than broad; yellowish brown with fine irregular growth lines and some blackish brown periostracal ridges, one of which runs from the apertural columellar border into the umbilicus. Peristome not reflected. Parietal, columellar and a short part of the adjoining basal apertural border thickened by a whitish callus. Most specimens with a continuous peristome and a narrow umbilical chink. Protoconch encrusted in all specimens; teleoconch without spiral sculpture.

##### Measurements.

(*N* = 9): H 2.2–2.4 mm, B 1.5–1.6 mm. Holotype 2.2×1.6 mm.

##### Ecology

**Figure 11.**
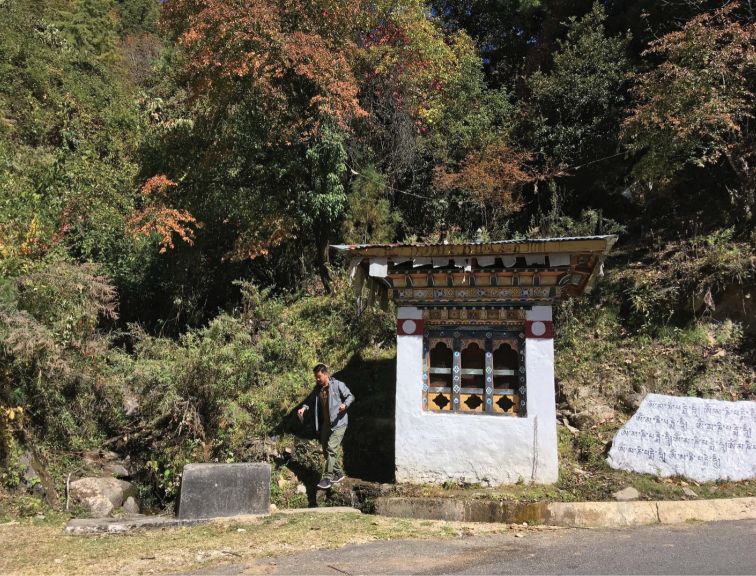
Type locality of both *Erhaia jannei* and *E. pelkiae*; Bhutan, district Thimphu, W of Geneykha, brooklet with water powered prayer wheel, 2750 m a.s.l. Photo by EG.

(Fig. 11). The species is uncommon on the rocks in the shaded streamlet uphill of the prayer wheel, where it occurs with *Galba truncatula* (Müller, 1774), *Physa* sp. (new for Bhutan), and *Erhaia pelkiae* sp. nov.

##### DNA data

(Fig. 18). The three individuals shared the same haplotype for both 16S rRNA (GenBank acc. no. MT239078) and COI (GenBank acc. no. MT237716). The uncorrected genetic p-distances between this species and *E. wangchuki* were 0.84% for 16S rRNA and 4.87% for COI. The distances were considerably higher when compared to *Erhaia* sp. from China, Guangxi, viz. 2.74% for 16S rRNA and 10.25% for COI (GenBank acc. nos. KC832722 and KC832701, respectively).

##### Notes.

This species was discovered in 2012, but since only a single shell was collected then, a description was considered premature.

##### Etymology.

The epithet *jannei* refers to Mr Janne Clewing, the son of the last author.

#### Erhaia pelkiae

Taxon classificationAnimaliaLittorinimorphaAmnicolidae

Gittenberger & Gyeltshen sp. nov.

819F33E6-9E01-5A96-978B-52B692112C7D

http://zoobank.org/8548A11B-5AAC-4D3E-9064-6321DF57677C

Figs 4 , 5 , 11

##### Material examined.

***Holotype*.** (Fig. 4) Bhutan • District Thimphu, c. 5 km E of Chhuzom, W of Geneykha; in brooklet with a prayer wheel along the road; 2750 m a.s.l.; 27°18'43"N, 89°36'10"E; E. Gittenberger, Choki Gyeltshen & Pema Leda leg. 25.X.2018; NBCB 1059. ***Paratype*.** (Fig. 5) 1 shell; same data as for holotype; NBCB 1060.

##### Diagnosis.

Shell with a partly reflected peristome, teleoconch with spiral lirae.

##### Shell.

Shell elongated ovoid, with 3½ convex, shouldered whorls that are separated by a deep suture; clearly higher than broad; light yellowish brown with fine growth lines and some brown periostracal ridges. Peristome reflected at the columellar and the basal side. Parietal and columellar side of the aperture thickened by a whitish callus. An irregular umbilical chink only in the paratype might be represent a malformation resulting from repair of the shell wall. Protoconch encrustated; teleoconch with fine spiral lines.

##### Measurements.

(*N* = 2): holotype and paratype H 1.9 mm, B 1.1 mm.

##### Ecology

(Fig. 11). The snails are rare on the rocks in the shaded streamlet uphill the prayer wheel. See also the data for *E. jannei*.

##### Notes.

The differences between the sympatric *E. pelkiae* and *E. jannei* are too large to regard as sexual dimorphism.

##### Etymology.

The epithet *pelkiae* refers to Ms. Pelki Yangdon, the daughter of the fourth author.

#### Erhaia wangchuki

Taxon classificationAnimaliaLittorinimorphaAmnicolidae

Gittenberger, Sherub & Stelbrink, 2017

636A6BE7-5F25-5DBF-8BD0-CC9B5C347E4A

Figs 6 , 7 , 12

Erhaia wangchuki Gittenberger, Sherub & Stelbrink, 2017a: 23 (“district Wangdue Phodrang, Gangchhu, 2883 m a.s.l.; 27°26'N, 90°11'E”). Gittenberger et al. 2017b: 43, fig. 28. Gittenberger et al. 2017c: 900, 903, figs 9, 10.

##### Material examined.

***Holotype*.** Bhutan • District Wangdue Phodrang, Gangchhu, 2883 m a.s.l.; 27°26'N, 90°11'E; Jigme Wangchuk leg. 21.III.2015; shell; NBCB1013. ***Paratypes*.** 2 shells; same data as for holotype; NBCB1014. Additional specimens from the type locality: 23 shells and 88 specimens in ethanol 70%, 10 specimens in ethanol 97%, E. Gittenberger, Choki Gyeltshen & Pema Leda leg. 22.X.2018; NBCB 1072.

##### Shell.

Shell conical, with 3–3½ convex, broadly shouldered whorls, that are separated by a deep suture; a little higher than broad; pale yellowish grey with fine irregular growth lines and some dark brown periostracal ridges, one of which sometimes running from a slightly angled site of the apertural columellar border into the umbilicus. Peristome not reflected. Parietal, columellar and about half the adjoining basal apertural border strongly thickened by a whitish callus. Most specimens with a continuous peristome and a broad umbilical chink. Protoconch with faint spiral lirae; teleoconch without spiral sculpture.

##### Measurements.

(*N* = 124): H 1.6–2.1 mm, B 1.3–1.7 mm.

##### Ecology

**Figure 12.**
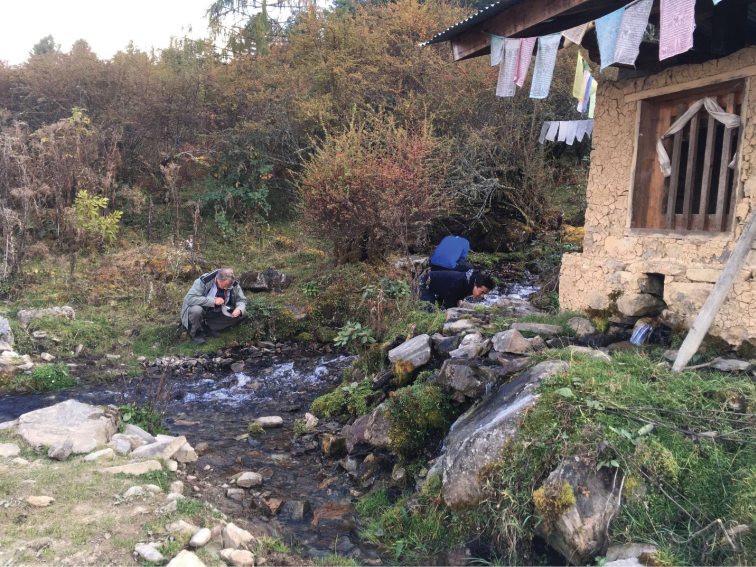
Type locality of *E. wangchuki*; Bhutan, district Wangdue Phodrang, Gangchhu, Gangzetem brooklet, 2883 m a.s.l. Photo by A.C. Gittenberger-de Groot.

(Fig. 12). See Gittenberger et al. (2017a) for data about the Gangzetem brooklet and its surroundings. The snails are very common on the pebbles and rocks in the open area near the road, next to the water powered prayer wheel.

##### DNA data

(Fig. 18). A single individual (GenBank acc. nos. KY798003 and MT237715, for 16S rRNA and COI, respectively) is genetically distinct from *E. jannei* (see data for that species) and showed genetic distances of 2.74% for 16S rRNA and 11.43% for COI compared to *Erhaia* sp. from China, Guangxi (GenBank acc. nos. KC832722 and KC832701).

##### Notes.

Only three relatively large shells form the type series of this species. Many more specimens, none of which exceed 1.7 mm in breadth and over 2.0 mm in height, were collected recently. This necessitated some adaptations in the description of the shells. Contrary to the original description, the shell should be described as higher than broad.

##### *Erhaia* in Nepal and northern India.

In their monograph on the aquatic molluscs of the Ganga River system Nesemann et al. (2007: 64–65) published short descriptions with drawings only of three *Erhaia* species from Nepal. We acquired photos of the holotypes of these nominal species, which are compared with the congeneric species from Bhutan and northern India.

#### Erhaia sugurensis

Taxon classificationAnimaliaLittorinimorphaAmnicolidae

Nesemann, Shah & Tachamo, 2007

BFB7EE71-4706-502F-B3A1-7F4E2A794F00

Fig. 10

Erhaia sugurensis Nesemann, Shah & Tachamo, 2007, in Nesemann et al. 2007: 65 (“Nepal, Central Zone, Lalitpur District, Godawari, upper reaches of Sugure Khola forrest stream, elevation of 1700 m a.s.l.”).

##### Shell.

According to Nesemann et al. (2007: 65) the shells are 1.6–1.9 mm high, with 3½ whorls that are “not convex”, the aperture is “rounded, widened and enlarged, the inner and outer lip is thickened”. The species is said to differ most conspicuously by “the separation of the last half whorl from the shell”.

##### Material examined

(photo only). ***Holotype*.** (NHMW 104172).

##### Discussion.

A flaring final half of the body whorl, though not as extreme as in the holotype of *E. sugurensis*, may also occur as an individual variation in *E. nainitalensis* and the width of the umbilical chink may vary, as is shown by Davis and Rao (1997: 277, figs 2A–F).

##### Notes.

*Erhaia sugurensis* occurs sympatrically with *E. banepaensis* at the type locality.

#### Erhaia banepaensis

Taxon classificationAnimaliaLittorinimorphaAmnicolidae

Nesemann & S. Sharma, 2007

17AF8006-2CC5-5BF8-B554-76DE58D31D70

Fig. 8

Erhaia banepaensis Nesemann & S. Sharma, in Nesemann et al. 2007: 64 (“Nepal, Central Zone, Kavre District, small forest stream, left tributary of the Chandeswari Khola upstream from Chandeshwari at Banepa”; the altitude is not indicated).

##### Shell.

The shells are described by Nesemann and Sharma (2007: 64) as 1.6–2.0 mm high, with 4–4½ “convex” whorls, an aperture that is “ovate but not widened and not enlarged”, with an inner lip that is “thin and fused to the body whorl”; it can be distinguished from the other Nepalese *Erhaia* species by the “conical and compact shape” and “convex” whorls (2007: 65).

##### Material examined

(photo only). ***Holotype*.** (NHMW 1033159).

##### Notes.

Nesemann and S. Sharma are mentioned as authors for this species, without specifying for what part of the text in Nesemann et al (2007) they have responsibility.

According to Nesemann et al. (2007: 64) *E. banepaensis* occurs sympatrically with *E. chandeshwariensis* at the shared type locality of these species. *Erhaia banepaensis* is supposed to be more widely distributed in Nepal between 1400 and 2085 m a.s.l., but the type series is restricted to the holotype (NHMW 103319 [not 1033159]) and a paratype (NHMW 103320). The location of the additional material is not indicated.

**Figure 13.**
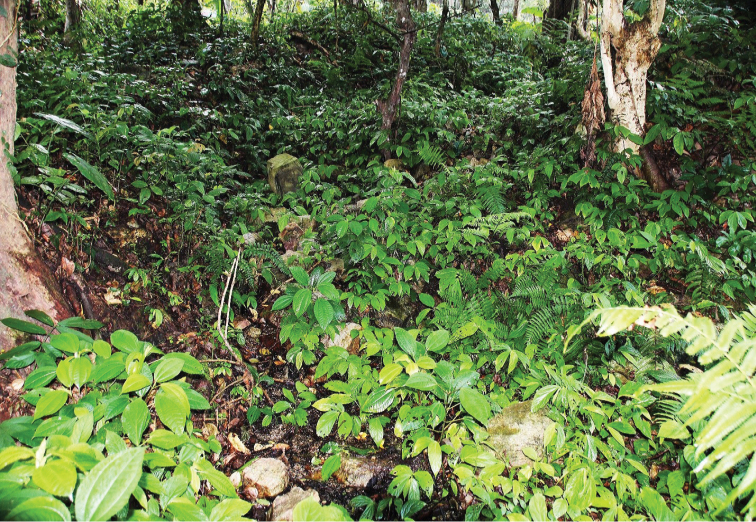
Habitat of *Tricula montana*; Bhutan, district Mongar, Chhuburee, 818 m a.s.l. Photo by JW.

#### Erhaia nainitalensis

Taxon classificationAnimaliaLittorinimorphaAmnicolidae

Davis & Rao, 1997

E2D86FBA-6588-57FC-9321-1203D47458A8

Fig. 9

Erhaia nainitalensis Davis & Rao, 1997: 276 (“India, Uttar Pradesh, Nainital District, Padampuiri”; “29°23'N, 79°30'E”)
Erhaia chandeshwariensis Nesemann and S. Sharma, in Nesemann et al. 2007: 64, 78 fig. 4 (“Nepal, Central Zone, Kavre District, small forest stream, left tributary of the Chandeswari Khola upstream from Chandeshwari at Banepa”; the altitude is not indicated).

##### Material examined

(photos only). Holotype and 4 paratypes of *Erhaia chandeshwariensis* (NHMW 103315 and 103316).

##### Discussion.

According to Davis and Rao (1997: 277, legends to figure 2) the holotype of *E. nainitalensis* is 2.28 mm high. However, elsewhere in the same article (Davis and Rao 1997: 279, table 1) the shell height of a single male animal is reported as 1.62 mm whereas 1.88–1.95–2.14 mm (minimum-mean-maximum) is indicated for five female snails. The difference in measurements between the single male and the five females might be indicative of sexual dimorphism. Shells of the species are also supposed to be “minute”, what is defined as 2.0 mm high or smaller (Davis and Rao 1997: 275, 289, table 5). We suppose that the dimensions of the holotype of *E. chandeshwariensis* , i.e. H 1.94 mm, fall within the range of the measurements of *E. nainitalensis*. Three of the four paratypes of *E. chandeshwariensis* (NHMW 103316/4) are c. 1.9 mm high, whereas the fourth shell is damaged, missing the apical whorls. Davis and Rao (1997: 278, fig. 3) figure a smooth columella for *E. nainitalensis*, but mention (p. 289) a “discernable glassy thickening of the columella”.

Judging the nominal taxa on the basis of photographs of shells and additional data in the literature, we conclude that in general shape and apertural characters, i.e. a narrow umbilical chink, a virtually smooth columella, and a thickened outer and inner lip, the holotype of *E. chandeshwariensis* cannot be distinguished from the shells of *E. nainitalensis* that are figured by Davis and Rao (1997: 277, figs 2A–F, 280, figs 4A–D). The fact that Davis and Rao (1997: 276) indicate 3¾-4 whorls for *E. nainitalensis*, whereas Nesemann and Sharma (2007: 64) mention 3½ whorls for *E. chandeshwariensis*, might be due to different counting methods.

###### Family Pomatiopsidae Stimpson, 1865

#### Genus Tricula

Taxon classificationAnimaliaLittorinimorphaPomatiopsidae

Benson, 1843

D99BC01B-5F17-555A-8AFD-B555B1CC6CB1

##### Type species by monotypy.

Melania (Tricula) montana Benson, 1843

#### Tricula montana

Taxon classificationAnimaliaLittorinimorphaPomatiopsidae

(Benson, 1843)

65E57C37-BD1F-572A-B522-BC1491C34D65

Figs 1 , 16–17

Melania (Tricula) montana Benson, 1843: 467 (“Bhimtal”, Nainital District, Uttarakhand, India; 1370 m a.s.l.). Lectotype in The Natural History Museum, London no. 1964426 (design. Davis et al. 1986: 428, fig. 3A).
Tricula montana ; Preston 1915: 68. Davis et al. 1986: 428–433, figs 3–4 (shell), 4 (operculum), 5–8 (anatomy), 9–10 (radula). Nesemann et al. 2007: 62, 78 pl. 15, fig. 1.

##### Material examined.

Bhutan • District Lhuentse: Khardungchhu; 27°31'56"N, 91°12'19"E; 1634 m a.s.l.; J. Wangchuk leg. 28.IV.2017; 3 shells; NBCB 1061. Same data except for 27.III.2019; 8 specimens in ethanol 70%; NBCB 1064.

District Lhuentse: Jarkangchhu; 27°32'27"N, 91°12'25"E; 1333 m a.s.l.; J. Wangchuk leg. 28.IV.2017; 2 shells; NBCB 1063. Same data except for 27.III.2019; 7 specimens in ethanol 70%; NBCB 1066.

District Lhuentse: Songkhangchhu; 27°31'54"N, 91°11'17"E; 1152 m a.s.l.; J. Wangchuk leg. 27.III.2019; 3 specimens in ethanol 70%; NBCB 1068.

District Lhuentse: Fawan; 27°29'22"N, 91°10'57"E; 940 m a.s.l.; J. Wangchuk leg. 27.III.2019; 3 specimens in ethanol 70%; NBCB 1069.

District Mongar: Chhuburee; 27°15'41"N, 91°09 02"E; 818 m a.s.l.; J. Wangchuk leg. 3.V.2017; 2 shells; NBCB 1062. Same data except for 26.III.2019; 5 specimens in ethanol 70%; NBCB 1065.

District Mongar: Rekpalung; 27°19'34"N, 91°13'28"E; 885 m a.s.l.; J. Wangchuk leg. 27.III.2019; 3 specimens in ethanol 70%; NBCB 1070.

District Trongsa: Chendebji; 27°29'24"N, 90°20'18"E; 2631 m a.s.l., J. Wangchuk photographed 12.I.2018 .

District Wangdue Phodrang: 40 km SSE of Wangdue Phodrang; 27°09'25"N, 90°04'05"E; 527 m a.s.l.; E. Gittenberger, Choki Gyeltshen & Kezang Tobgay leg. 24.IX.2019; 23 shells; 23 specimens in ethanol 70%; 10 specimens in ethanol 97%; NBCB 1084.

District Zhemgang, Kekhar, 27°12'37"N, 90°46'28"E; 1540 m a.s.l., J. Wangchuk leg. photographed 17.I.2018.

##### Shell.

Shell slender conical, with up to c. 5 shouldered, moderately convex whorls, separated by an incised suture; with obsolete growth lines and poorly discernible dense spiral lirae. Pale yellowish grey, with a light brown apertural border when fully grown. Apex not flattened, often decollate. Aperture triangular with broadly rounded edges, its parietal side about double the length of the columellar side; palatal side straight, passing into the slightly curved basal border with a more strongly curved transitional part. Parietal border of the aperture attached, at least in the middle and not or only slightly protruding. Umbilicus closed or nearly so.

##### Measurements.

According to Davis et al. (1986: 431) the shell height of males and females combined (*N* = 10) is 3.32–3.72 mm. However, for the lectotype a larger shell height is indicated, i.e. 3.92 mm (Davis et al. 1986: 429, fig. 3A, 430). That shell is not even a relatively large specimen. Additional shells figured by Davis et al. (1986: 429, 430, fig. 3F, G, H, K, L) and printed at the same scale, are larger.

The shells that are known from Bhutan (*N* = 73) are relatively small, with 5–5½ whorls measuring H 2.8–3.6 mm, B 1.3–1.7 mm.

##### Distribution.

(Fig. 1). According to Subba Rao (1989: 68) this species occurs in the Indian states of Himachal Pradesh (“Jhiri valley”) and Uttarakhand (= Uttaranchal). Nesemann et al. (2007: 62) refers to it as widely distributed in the western and central Himalaya, in Nepal mainly at 1300–2100 m a.s.l. The records for Bhutan, at altitudes of 527–2631 m a.s.l., extend its range eastwards.

##### Habitat.

**Figure 14.**
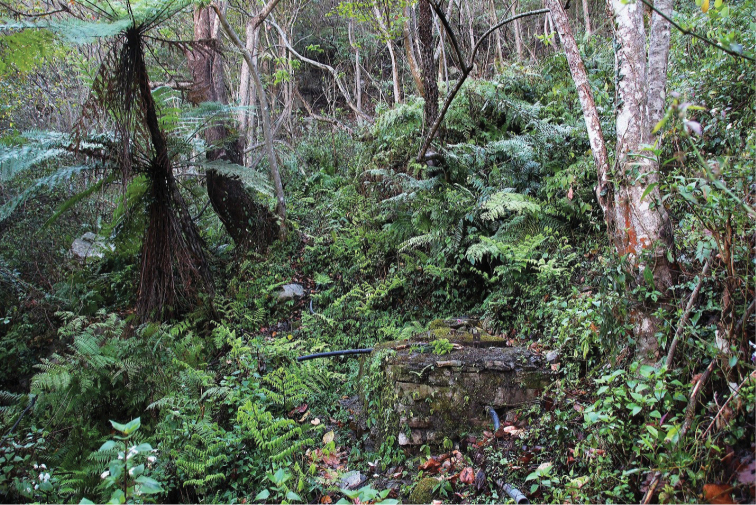
Habitat of *Tricula montana*; Bhutan, district Lhuentse, Jarkangchhu, 1333 m a.s.l. Photo by JW

**Figure 15.**
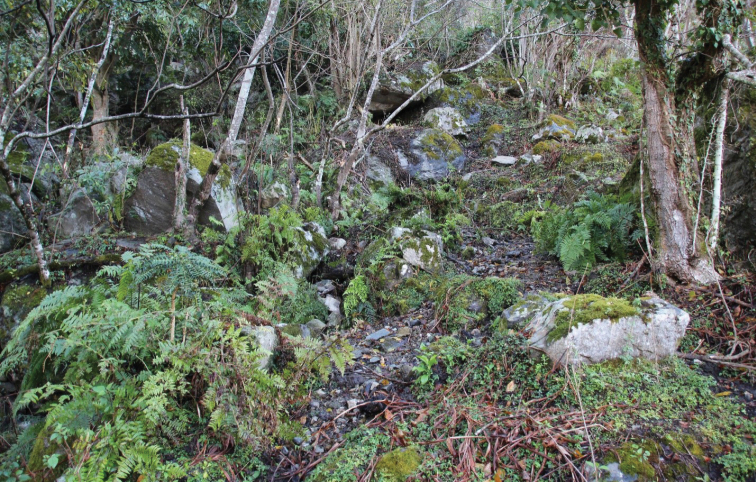
Habitat of *Tricula montana*; Bhutan, district Lhuentse, Khardungchhu, 1634 m a.s.l. Photo by JW.

This species was found in Bhutan without accompanying *Erhaia* species mostly in densely vegetated, shaded areas with more or less overgrown springs and streamlets (Figs 1, 13–15). Davis et al. (1986: 427) describe a similar habitat for the Nainital District near the type locality of *T. montana*. The locality in the district of Wangdue Phodrang is an overgrown, dripping wet, vertical, rocky wall along the road.

**Figures 16, 17.**
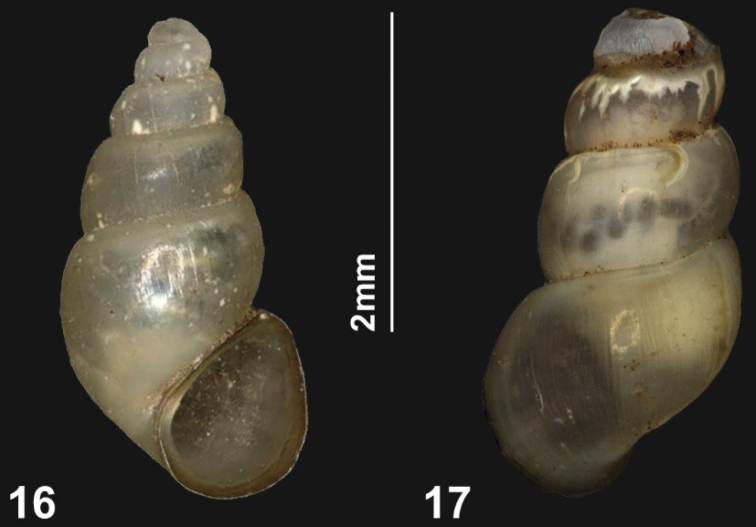
*Tricula montana* (Benson, 1843), Bhutan **16** District Lhuentse, Khardungchhu, 1634 m a.s.l. (H 3.1 mm) **17** Bhutan, district Mongar, Chhuburee, 818 m a.s.l. (decollate specimen, H 3.0 mm). Photos by J. Goud.

The shells from Mongar, Chhuburee, and from Lhuentse, Jarkangchhu, are all decollate (Fig. 17), whereas shells from the other localities still have their apical whorls present (Fig. 16). This might be a consequence of unknown differences in water quality at those different localities.

##### DNA data.

Two snails from Chhuburee and two snails from Khardungchhu were sequenced. These specimens shared the same haplotype per population for both 16S rRNA (GenBank acc. nos. MT239080 and MT239079, for Chhuburee and Khardungchhu,) and COI (GenBank acc. nos. MT237718 and MT237717, for Chhuburee and Khardungchhu). The two populations differed genetically by 1.0% and 4.9% for 16S rRNA and COI, respectively. Because the monophyly of *Tricula* remains uncertain (see e.g., Liu et al. 2014), we compared these sequences with additional data available from GenBank. For 16S rRNA, the lowest genetic distances, i.e., 3.1% and 3.3%, were identified between snails from Chhuburee and Khardungchhu, respectively, and *Tricula* sp. from China, Hunan, Xiangxi, Fenghuang (GenBank acc. no. EU311736), and 3.3% and 3.5% between snails from Chhuburee and Khardungchhu, respectively, and *T. ludongbini* Davis & Y.-H. Guo, 1986 from China, Yunnan, Panlong River, Hei Long Tan (GenBank acc. no. KC832717).

The genetic distances between *T. montana* from Chhuburee and Khardungchhu were considerably higher for COI, with 8.9% and 9.3%, respectively, between snails from Chhuburee and Khardungchhu and *Tricula* sp. from China, Sichuan, Dayi, Tian Gong Mia, Huang Ba (GenBank acc. no. AF253070), and *Tricula hortensis* Attwood & Brown, 2003 from China (GenBank acc. no. JQ082621).

##### Notes.

The species was identified conchologically by using the data provided by Benson (1843), Davis et al. (1986) and Nesemann et al. (2007), taking also the distributional data (Nesemann et al. 2007: 62) into account.

Some species of *Tricula* may transmit schistosomes that could in principle infect humans and other mammals. No data in respect of this are known for *T. montana*.

See Davis et al. (1986) for a detailed account on this species, with data on shell morphology, anatomy of males and females, biogeography, and systematic relationships.

**Figure 18.**
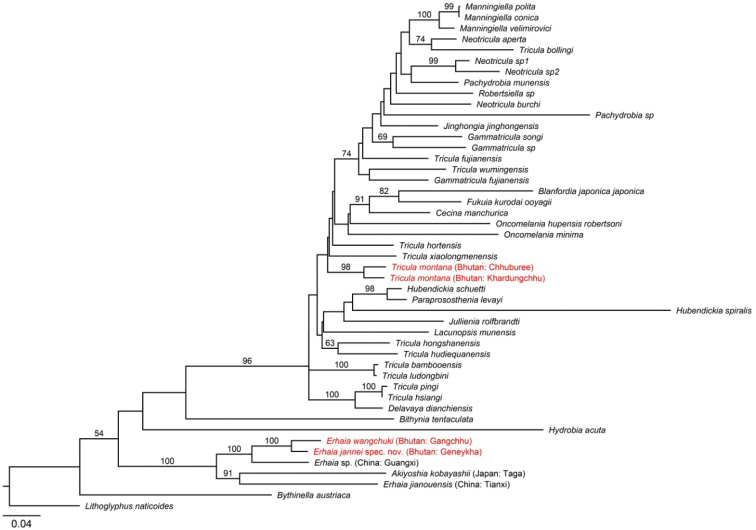
Maximum likelihood tree reconstructed with RAxML BlackBox (Stamatakis et al. 2008; GTR+G substitution model for each partition and 100 bootstrap replicates) based on the 16S rRNA and COI dataset of Liu et al. (2014) and Guan et al. (2008), with new data in red. Numbers on branches denote bootstrap values >50.

## Supplementary Material

XML Treatment for Erhaia

XML Treatment for Erhaia jannei

XML Treatment for Erhaia pelkiae

XML Treatment for Erhaia wangchuki

XML Treatment for Erhaia sugurensis

XML Treatment for Erhaia banepaensis

XML Treatment for Erhaia nainitalensis

XML Treatment for Tricula

XML Treatment for Tricula montana
